# Response to Ustekinumab Therapy Is Associated with an Improvement of Nutritional Status in Patients with Crohn’s Disease †

**DOI:** 10.3390/jcm12196118

**Published:** 2023-09-22

**Authors:** Lorenzo Bertani, Claudia D’Alessandro, Marco Fornili, Francesca Coppini, Federico Zanzi, Luca Carmisciano, Francesca Geri, Giovanni Baiano Svizzero, Emma Maria Rosi, Alice De Bernardi, Linda Ceccarelli, Maria Gloria Mumolo, Laura Baglietto, Massimo Bellini, Nicola De Bortoli, Francesco Costa

**Affiliations:** 1 Department of Translational Research and New Technologies in Medicine and Surgery, University of Pisa, Via Roma, 67, 56100 Pisa, Italy; dalessandroclaudia@gmail.com (C.D.); coppini.francesca@gmail.com (F.C.); federico.zanzi@outlook.com (F.Z.); francesca.geri@libero.it (F.G.); emma.maria.rosi@gmail.com (E.M.R.); alice.debernardi@outlook.com (A.D.B.); massimo.bellini@unipi.it (M.B.); nicola.debortoli@unipi.it (N.D.B.); 2 Tuscany North West ASL, Department of General Surgery and Gastroenterology, Pontedera Hospital, Via Roma, 147, 56025 Pontedera, Italy; 3 Department of Clinical and Experimental Medicine, University of Pisa, Via Roma, 67, 56100 Pisa, Italy; marco.fornili@unipi.it (M.F.); l.carmisciano@studenti.unipi.it (L.C.); laura.baglietto@unipi.it (L.B.); 4 Department of General Surgery and Gastroenterology, IBD Unit, Pisa University Hospital, Via Paradisa, 2, 56124 Pisa, Italy; g.baianosvizzero@gmail.com (G.B.S.); ceccarellilinda@gmail.com (L.C.); g.mumolo@int.med.unipi.it (M.G.M.); fcosta@med.unipi.it (F.C.)

**Keywords:** Crohn’s disease, sarcopenia, ustekinumab, biologics, BIA

## Abstract

The presence of sarcopenia has been associated with the worst outcome of Crohn’s disease (CD). At present, no studies have evaluated the impact of ustekinumab (UST) in terms of its effects on body composition. The aim of this prospective study was to evaluate whether UST treatment could modify the parameters of body composition as assessed by bioelectrical impedance assay (BIA) in patients with CD. We prospectively enrolled consecutive patients with CD treated with UST, evaluating the therapeutic outcome at week 48 in terms of clinical remission and mucosal healing. BIA was performed at baseline and at week 48, assessing body cellular mass, total body water, phase angle, and body mass index. Out of 44 patients enrolled, 26 (59%) were in clinical remission and 22 (50%) achieved mucosal healing at the end of follow up. No significant differences were observed at baseline in all the BIA parameters between responders and non-responders. Phase angle increased over time in responders, while this was not observed in non-responders (test for the interaction between time and outcome, *p*-value = 0.009 and 0.007 for clinical remission and mucosal healing, respectively). The same differential increase was observed for body cellular mass (test for the interaction between time and outcome, *p*-value = 0.03 and 0.05 for clinical remission and mucosal healing, respectively). Total body water and BMI increased homogenously over time regardless of the outcomes (tests for the association with time, *p*-values of 0.01). To conclude, responsiveness to UST therapy seems to be associated with body composition modifications in patients with CD. In particular, the increase in phase angle in responders suggests that a significant improvement of nutritional status occurred in these patients.

## 1. Introduction

Crohn’s disease (CD) is a chronic inflammatory disease characterized by abdominal pain, diarrhea, endoscopic inflammation, and malnutrition. Indeed, several studies showed that intestinal malabsorption and catabolic effects of acute inflammation can induce weight loss and malnutrition with an incidence ranging from 25 to 80% [1,2,3]. Other important factors leading to malnutrition in patients with CD are reduced oral food intake, bacterial overgrowth, impaired epithelial transport, and the loss of epithelial integrity [4].

Malnutrition could be characterized by the deficiency of vitamins or micronutrients, but also by the depletion of proteins. However, it is worth noting that one of the most important aspects of malnutrition in CD is correlated with the alterations of body composition, which could have an important impact on the responsiveness to medical and surgical treatments, as well as on the disease course [5]. The most important modification of body composition in patients with CD is the reduction of lean muscle mass, a condition defined as sarcopenia. Notably, the presence of sarcopenia is particularly important for the disease course and could be present even under disease remission [6], with an important impact even on quality of life of patients with CD.

The best way to assess body composition and, consequently, to have an indirect measurement of nutritional status, is bioelectrical impedance analysis (BIA), which is widely used in several chronic diseases involving metabolic derangements [7]. BIA is a non-invasive tool able to measure whole-body impedance, which is the opposition of the body to alternating current consisting of two components: resistance (R) and reactance (Xc). The most clinically relevant BIA-derived parameter is the phase angle (PhA), an index of cell membrane integrity and vitality, which is able to provide crucial information on cellular health and soft tissue hydration. The PhA is considered to be a strong predictor of survival in several diseases [8,9,10,11], including inflammatory bowel diseases (IBD) [12,13].

Surprisingly, despite the high rates and the important impact of malnutrition and sarcopenia in patients with CD [14], nutritional assessment has always had a marginal role in their management [15]. Some studies have been performed to evaluate the impact of anti-TNF therapies in terms of the improvement of BIA-derived parameters, but no studies have ever been performed in patients with CD treated with ustekinumab (UST). Interestingly, Galluzzo et al. [16] showed that UST therapy could be able to increase PhA in patients with psoriasis, but no data are currently available for patients with inflammatory bowel diseases. Nevertheless, clinical trials and real-life studies showed that UST is effective in inducing clinical remission and mucosal healing in CD and in ulcerative colitis [17,18,19], and, therefore, it is currently one of the most used treatment options in these settings.

Based on the above background, the aim of the present study was to assess whether responsiveness to UST therapy is associated with body composition modification in patients with CD, evaluating the therapeutic outcome in terms of mucosal healing and the nutritional status in terms of PhA.

## 2. Materials and Methods

We included in this prospective study all consecutive patients with CD who started a biological treatment with UST as monotherapy at the Gastroenterology Unit of Pisa University Hospital, according to ECCO guidelines [20], from the start of September 2020 to the end of September 2021. Patients treated concomitantly with azathioprine or other immunosuppressants, albeit for other immune-mediated diseases, were excluded from the study.

The study was conducted in full compliance with the Declaration of Helsinki and was approved by the Ethics Committee of Pisa University Hospital (CEAVNO) on 23 July 2020. All patients provided their informed consent to the collection and publication of data.

We collected the following data at baseline: age, sex, disease extent according to Montreal classification, previous biological treatments, previous intestinal or perianal surgery, and disease severity according to the Harvey Bradshaw Index (HBI) in combination with the Simple Endoscopic Score for CD (SES-CD).

An expert team of clinicians evaluated patients at baseline and at week 48, assessing the disease clinical activity in terms of HBI and evaluating therapeutic response. At baseline, at week 24, and at week 48, we collected fecal calprotectin (FC) levels.

At baseline and at week 48, every patient performed BIA in order to estimate body composition in terms of body cellular mass (BCM), total body water (TBW), body mass index (BMI), R, and Xc. BIA allows the assessment of several parameters by measuring the impedance offered by the body to the passage of an alternating electric current flow at low intensity (800 µA) and fixed frequency. Since the administered current flows predominantly through low-resistance materials and fat-free mass, seeking the path of the lowest resistance, there is a lag in the current, which leads to a phase shift [21]. This phase shift can be represented geometrically as an angular transformation of the ratio of reactance to resistance: this is known as the PhA [21]. Mathematically, PhA was calculated according to this formula: PhA = arctan (Xc/R)∙180/π [21]. BIA assessments were performed by using a Bioelectrical Impedance single frequency Analyzer (BIA/STA, Akern^®^, Florence, Italy) with a distal, tetra polar technique, delivering an excitation current at 50 kHz.

All of the enrolled patients underwent a colonoscopy at both baseline and week 48, when mucosal healing (defined as the disappearance of ulcers) was assessed. All the endoscopists were experts in the evaluation of CD endoscopic scores and were blinded from BIA parameters, as well as FC levels at baseline and at week 24. At week 48, clinical remission was defined as an HBI < 5 without concomitant corticosteroid therapy.

**Figure 1:**
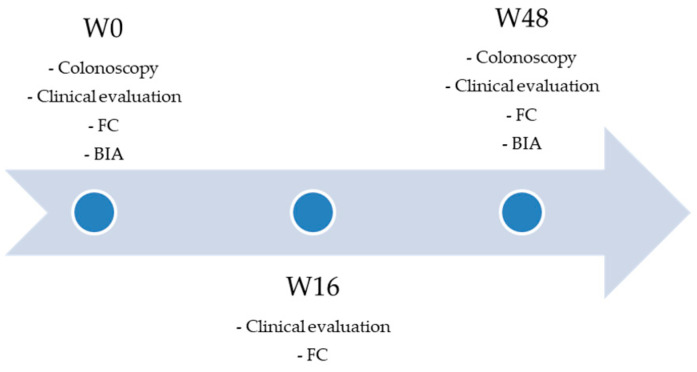
Outline of the study.

The study is schematized in Figure 1.

### Statistical Analysis

Continuous variables were summarized with medians and interquartile ranges (IQRs), categorical variables with counts, and percentages. We applied mixed effect models with patients fitted at random levels to describe the trend of anthropometric variables; the models included time, the outcome (either clinical remission or mucosal healing), and their interactions as explanatory variables. FC levels were log-transformed to achieve a nearly normal distribution. Predicted values and *p*-values of the associations were reported. We refitted models with an interaction term’s *p*-value above 0.05, removing the interaction term to estimate the association of anthropometric variables and FC with time and outcomes. The within-subject changes of the anthropometric measurements and FC levels over time in responders and non-responders were visualized through box-plots.

All statistical analyses were performed using R software, version 4.2.2 (R Core Team (2022). R: A language and environment for statistical computing. R Foundation for Statistical Computing, Vienna, Austria. URL: https://www.R-project.org/ (accessed on 10 July 2023)).

## 3. Results

The study sample included 44 patients with CD, of which 26 (59%) were in clinical remission and 22 (50%) showed mucosal healing at the end of follow up (week 48). Only two patients were naïve to any biological therapy, while 42 were previously treated with at least one anti-TNF drug.

Table 1 reports the baseline characteristics of the patients, overall and by response to the treatment. There was no evidence of association of BIA parameters or FC at baseline with response to therapy.

**Figure 2:**
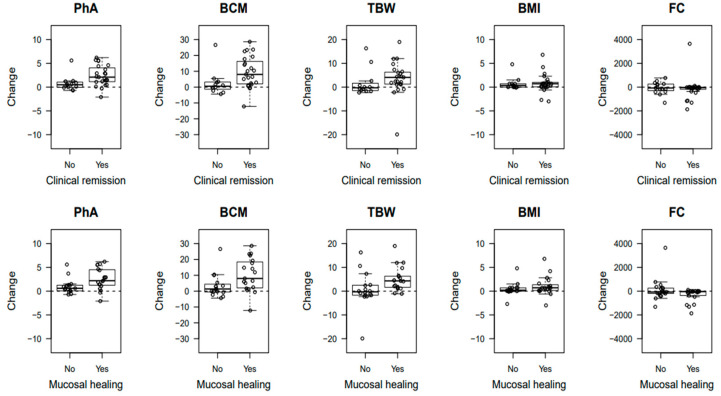
Box-plots of the changes in phase angle (PhA), body cell mass (BCM), total body water (TBW), body mass index (BMI), and fecal calprotectin (FC) at 48 weeks with respect to baseline by response to treatment.

Figure 2 shows the box-plots of the changes of BIA variables and FC at 48 weeks with respect to the baseline by outcome.

The analyses of the trends of anthropometric parameters and biomarkers by time and outcome are reported in Table 2.

PhA increased over time in those responding to the treatment, but not in those not responding (test for the interaction between time and outcome, *p*-value of 0.009 and 0.007 for clinical remission and mucosal healing, respectively). The same differential increase was observed for BCM (test for the interaction between time and outcome, *p*-value of 0.03 and 0.05 for clinical remission and mucosal healing, respectively). TBW and BMI increased homogenously over time regardless of the outcomes (tests for the association with time, *p*-values of 0.01).

As concerns FC, responders’ levels were lower (test for the association with the outcome, *p*-value of 0.03 and 0.05 for clinical remission and mucosal healing, respectively) and decreased over time, whereas, in non-responders, the results were higher and stable over time (test for the interaction between time and outcome, *p*-value of 0.02 and <0.001 for clinical remission and mucosal healing, respectively).

## 4. Discussion

The present study was designed to evaluate the possible effect of UST therapy on body composition in patients with CD. Our results highlighted that patients responding to UST treatment, both in terms of clinical remission and mucosal healing, increase their values of PhA and BCM, assessed by BIA, in only one year of treatment. To the best of our knowledge, this is the first report of this important finding.

Malnutrition is one of the most common complications of IBD, since up to 80% of patients can develop it throughout their lives. Significantly, a greater degree of malnutrition can be seen in patients with CD compared to UC, mainly due to small intestine involvement, which can induce malabsorption [22]. Nevertheless, several other different factors could account for the development of malnutrition in CD, including the reduced oral food intake, bacterial overgrowth [23], the use of corticosteroids [24], and surgical resections [4]. The disease extension and its duration are important factors in determining the severity of malnutrition, but a key role is played by the inflammatory systemic response mediated by pro-inflammatory cytokines, such as tumor necrosis factor (TNF) and interleukins-1 and -6, which can increase catabolism and lead to anorexia [25]. The presence of malnutrition, especially in case of sarcopenia, is clearly associated with worse outcomes of IBD. In particular, some data showed an increased risk of surgery [26] and surgical comorbidities [27], as well as a higher rate of failure anti-TNF therapies [28].

According to the ESPEN definition of malnutrition as “a state resulting from the lack of intake or uptake of nutrition that leads to altered body composition and body cell mass leading to diminished physical and mental function and impaired clinical outcome from disease” [29], a reliable assessment of body composition is crucial. BIA is able to estimate the body composition of patients regardless of BMI [30], and, for this reason, it has been compared to the radiologic assessment of lean muscle mass in the detection of sarcopenia, obtaining similar results [31]. Moreover, this technique is a more comprehensive evaluation of nutritional status, and it is widely used in several chronic diseases leading to metabolic derangements [7]. With regard to IBD, the nutritional markers evaluated by using BIA have been correlated with serum nutritional markers and inversely correlated with disease activity, both in adults [32] and in children [12]. BIA is able to estimate the body composition through the administration of low-intensity electric current, which flows through the body at different rates in relation to its composition [33]. The living cells store the electric charge flowing in the current and causes a delay in the flow, like a tiny capacitor. This capacitive resistance, or reactance, depends on the composition and integrity of the BCM, defined as the metabolically active part of the body [21]. Conversely, the body fat or the dead cells resist the flow of electric current. PhA is the more reliable BIA-derived parameter in defining nutritional status, and it is considered as a strong predictor of survival in several diseases [8,9,10,11], and, concerning IBD, it has been associated with nutritional deficiencies [12,13]. Recently, a study by Peng et al. [34] showed that PhA is strongly associated with malnutrition, with high reliability, independently from hematic parameters, such as serum albumin, in patients with CD.

Despite the high rates of malnutrition in CD, few studies evaluated this outcome during biological therapy. Subramaniam et al. [35] showed that infliximab therapy increased lean muscle mass, evaluated by magnetic resonance imaging, in a small cohort of patients with CD after 25 weeks of treatment. Subsequently, a comparative study by Emerenziani et al. [33] showed that patients with CD significantly increased their PhA after the induction of infliximab therapy. Notably, this study even highlighted that patients in maintenance treatment with infliximab had significantly higher levels of PhA in comparison with those on conventional therapy, but the latter group displayed lower rates of disease remission [36]. This could be intended as a confirmation of the high importance of inflammatory status in the pathogenesis of sarcopenia. In our cohort, patients with CD responding to UST therapy significantly increased their PhA, highlighting that this drug could improve their nutritional status. The same differential increase was observed even for BCM, although it reached statistical significance only when evaluating responders in terms of clinical remission. Our results are in line with those from Galluzzo et al. [16], which showed that UST therapy increased PhA as well as BCM in patients with psoriasis. This is particularly important in this disease, since its activity is correlated strictly and directly to the percentage of fat mass [37,38]. However, the impact of malnutrition in terms of long-term disease outcomes is significantly more important in CD than in psoriasis.

The present study also showed that FC levels at week 24 were significantly lower in patients achieving clinical and endoscopic remission at week 48. This is in line with the results of a post-hoc analysis of UNITI/IM-UNITI studies, where week 6 FC levels <250 mg/kg were associated with endoscopic remission at week 52 [39]. We previously demonstrated the reliability of FC after the induction of biological therapies with infliximab, adalimumab, golimumab, and vedolizumab [40], but, to the best of our knowledge, this is the first evidence of the predictive value of FC in patients with CD treated with UST in a real-life setting. This is a relevant point, since real life patients are often more complex than those included in clinical trials, but the everyday clinical practice should be based on real-life data [41]. Baseline FC median levels were particularly low in both responders and non-responders, probably due to the inclusion of several patients with ileal involvement. Indeed, FC is frequently lower in patients with ileal CD in comparison with patients with ulcerative colitis [42,43]. It is worth noting that baseline FC levels were similar both in patients who achieved mucosal healing at week 48 and in non-responders, and this should be interpreted as a signal of homogeneity of the cohort, thus avoiding selection bias. Obviously, at week 48, the responders displayed FC levels significantly lower than non-responders.

The major point of strength of the present study is that this is the first investigation providing evidence of the efficacy of UST in CD in terms of improvement of nutritional status. We are now in an era with a rapidly expanding therapeutic armamentarium for patients with CD; thus, the choice of the right drug should take into consideration nutritional outcomes in addition to clinical and endoscopic ones. Moreover, the prospective design and the evaluation of the therapeutic endpoint in terms of mucosal healing undoubtedly increased the significance of our results. Indeed, according to current guidelines, the therapeutic target in CD should always be assessed by endoscopy. Finally, our results confirmed the reliability of FC in predicting therapeutic efficacy.

The present study also has some limitations. Above all, the limited sample size could not allow us to obtain definitive conclusions, although the statistical significance was reliably achieved. Moreover, the assessment of lean muscle mass by magnetic resonance imaging would improve the significance of the study; however, it is worth noting that BIA is widely considered comparable to radiologic tests in defining the presence of sarcopenia [28]. Lastly, the presence of a control group of patients treated with another biological therapy could allow for a better understanding of the real impact of UST in improving the body composition.

## 5. Conclusions

In conclusion, in the present study, we have provided evidence that UST therapy is able to improve the nutritional status in patients with CD. In the future, larger studies could confirm these findings in order to better understand the role of this treatment and its role in modifying body composition.

## Figures and Tables

**Table 1:** Baseline characteristics of patients, overall and by response to the treatment.

	All	Clinical Remission			Mucosal Healing		
		No	Yes	*p*-Value *	No	Yes	*p*-Value *
	*n* = 44	*n* = 18 (41%)	*n* = 26 (59%)		*n* = 22 (50%)	*n* = 22 (50%)	
*Sex*, *n* (%)				*0.75*			*1.00*
Female	17 (39)	6 (33)	11 (42)		9 (41)	8 (36)	
Male	27 (61)	12 (67)	15 (58)		13 (59)	14 (64)	
*Age*	49 (36, 55)	52 (36, 58)	47 (36, 54)	*0.44*	51 (36, 58)	46 (36, 54)	*0.53*
*Montreal L at baseline*, *n* (%)				*0.96*			*0.96*
L1	10 (23)	5 (28)	5 (19)		6 (27)	4 (18)	
L2	10 (23)	4 (22)	6 (23)		5 (23)	5 (23)	
L3	22 (50)	8 (44)	14 (54)		10 (45)	12 (55)	
L3 + L4	2 (5)	1 (6)	1 (4)		1 (5)	1 (5)	
*Montreal B at baseline*, *n* (%)				*0.96*			*0.97*
B1	8 (18)	3 (17)	5 (19)		4 (18)	4 (18)	
B1 + B2	1 (2)	1 (6)	0 (0)		1 (5)	0 (0)	
B1p	2 (5)	1 (6)	1 (4)		1 (5)	1 (5)	
B2	10 (23)	4 (22)	6 (23)		5 (23)	5 (23)	
B2 + B3	6 (14)	2 (11)	4 (15)		2 (9)	4 (18)	
B2 + B3p	11 (25)	4 (22)	7 (27)		6 (27)	5 (23)	
B2p	3 (7)	1 (6)	2 (8)		1 (5)	2 (9)	
B3p	3 (7)	2 (11)	1 (4)		2 (9)	1 (5)	
*Previous intestinal surgery*, *n* (%)				*0.36*			*1.00*
No	20 (45)	10 (56)	10 (38)		10 (45)	10 (45)	
Yes	24 (55)	8 (44)	16 (62)		12 (55)	12 (55)	
*Previous perianal surgery*, *n* (%)				*1.00*			*1.00*
No	26 (59)	11 (61)	15 (58)		13 (59)	13 (59)	
Yes	18 (41)	7 (39)	11 (42)		9 (41)	9 (41)	
*PhA at baseline*, *median (IQR)*	5.20 (4.20, 6.60)	4.80 (3.80, 6.60)	5.60 (4.75, 6.57)	*0.39*	4.90 (4.00, 6.50)	5.60 (4.78, 6.60)	*0.42*
*BCM at baseline*, *median (IQR)*	25.6 (20.9, 29.4)	25.2 (22.2, 36)	25.9 (20.8, 28.5)	*0.75*	25.2 (20.9, 29.3)	26.3 (20.3, 29.2)	*0.94*
*TBW at baseline*, *median (IQR)*	35.2 (32, 42.9)	37.1 (32.1, 42.9)	34.7 (30.8, 42.7)	*0.81*	35.2 (32.1, 42.1)	35.0 (31.1, 43.7)	*0.92*
*BMI at baseline*, *median (IQR)*	22.7 (19.6, 24.5)	22.6 (19.6, 24.6)	22.8 (20.8, 24.3)	*0.81*	22.9 (19.6, 24.6)	22.6 (19.9, 24.3)	*0.98*
*FC at baseline*, *median (IQR)*	210 (62, 586)	330 (210, 825)	152 (50, 366)	*0.06*	292 (183, 634)	96 (44, 502)	*0.10*

* Fisher’s exact test for the categorical variables and Mann–Whitney’s test for the continuous variables. Number of missing data: PhA at baseline *n* = 1; BCM at baseline *n* = 1; BCM at week 48 *n*; TBW at baseline *n* = 1; BMI at baseline *n* = 1; FC at baseline *n* = 1. Abbreviations: PhA: phase angle; BCM: body cell mass; TBW: total body water; BMI: body mass index; FC: fecal calprotectin.

**Table 2:** Trend of BIA parameters and fecal calprotectin by time and outcomes.

Clinical Remission									
	No			Yes		Time	CR	Time × CR	
	Baseline		w48	Baseline	w48	*p*-Value	*p*-Value	*p*-Value	
PhA (Ω)	5.36		5.86	5.72	8.15	*0.37*	*0.58*	*0.009*	
BCM (Kg)	28.1		30.0	26.1	35.5	*0.48*	*0.54*	*0.03*	
TBW (l)	37.4		40.3	37.6	40.5	*0.01*	*0.95*	*-*	
BMI (kg/m^2^)	23.5		24.3	22.8	23.6	*0.01*	*0.64*	*-*	
	**Baseline**	**w24**	**w48**	**Baseline**	**w24**	**w48**	***p*-value**	***p*-value**	***p*-value**
FC (mg/Kg)	321	318	316	133	83	52	*0.96*	*0.03*	*0.02*
**Mucosal healing**									
	**No**			**Yes**		**Time**	**MH**	**Time × MH**	
	**Baseline**		**w48**	**Baseline**	**w48**	***p*-value**	***p*-value**	***p*-value**	
PhA (Ω)	5.32		6.06	5.82	8.45	*0.13*	*0.43*	*0.007*	
BCM (Kg)	27.1		30.3	26.8	36.4	*0.17*	*0.92*	*0.05*	
TBW (l)	36.8		39.7	38.2	41.1	*0.01*	*0.58*	*-*	
BMI (kg/m^2^)	23.2		24.0	23.1	23.9	*0.01*	*0.95*	*-*	
	**Baseline**	**w24**	**w48**	**Baseline**	**w24**	**w48**	***p*-value**	***p*-value**	***p*-value**
FC (mg/Kg)	280	293	307	128	70	39	*0.72*	*0.05*	*<0.001*

Predicted values from the mixed effect model including linear time, clinical remission, their interactions as predictors, and patients fitted at random levels. Where the interaction term significance was above 0.05, the mixed model included only time and clinical remission as predictors and patients at random levels. CR: clinical remission; Time × CR: significance of the interaction term between clinical remission and time; MH: mucosal healing; Time × MH: significance of the interaction term between mucosal healing and time; PhA: phase angle; BCM: body cell mass; TBW: total body water; BMI: body mass index; FC: fecal calprotectin.

## Data Availability

Data are available upon request to corresponding author.

## References

[1] Geerling B.J., Badart-Smook A., Stockbrugger R.W., Brummer R.J. (2000). Comprehensive nutritional status in recently diagnosed patients with inflammatory bowel disease compared with population controls. Eur. J. Clin. Nutr..

[2] Baumgart D.C., Sandborn W.J. (2007). Inflammatory bowel disease: Clinical aspects and established and evolving therapies. Lancet.

[3] Peyrin-Biroulet L., Loftus E.V., Colombel J.F., Sandborn W.J. (2011). Long-term complications, extraintestinal manifestations, and mortality in adult Crohn’s disease in population-based cohorts. Inflamm. Bowel Dis..

[4] Balestrieri P., Ribolsi M., Guarino M.P.L., Emerenziani S., Altomare A., Cicala M. (2020). Nutritional Aspects in Inflammatory Bowel Diseases. Nutrients.

[5] Bryant R.V., Trott M.J., Bartholomeusz F.D., Andrews J.M. (2013). Systematic review: Body composition in adults with inflammatory bowel disease. Aliment. Pharmacol. Ther..

[6] Valentini L., Schaper L., Buning C., Hengstermann S., Koernicke T., Tillinger W., Guglielmi F.W., Norman K., Buhner S., Ockenga J. (2008). Malnutrition and impaired muscle strength in patients with Crohn’s disease and ulcerative colitis in remission. Nutrition.

[7] Marra M., Sammarco R., De Lorenzo A., Iellamo F., Siervo M., Pietrobelli A., Donini L.M., Santarpia L., Cataldi M., Pasanisi F. (2019). Assessment of Body Composition in Health and Disease Using Bioelectrical Impedance Analysis (BIA) and Dual Energy X-Ray Absorptiometry (DXA): A Critical Overview. Contrast Media Mol. Imaging.

[8] Beberashvili I., Azar A., Sinuani I., Shapiro G., Feldman L., Stav K., Sandbank J., Averbukh Z. (2014). Bioimpedance phase angle predicts muscle function, quality of life and clinical outcome in maintenance hemodialysis patients. Eur. J. Clin. Nutr..

[9] Han B.G., Lee J.Y., Kim J.S., Yang J.W. (2018). Clinical Significance of Phase Angle in Non-Dialysis CKD Stage 5 and Peritoneal Dialysis Patients. Nutrients.

[10] Grundmann O., Yoon S.L., Williams J.J. (2015). The value of bioelectrical impedance analysis and phase angle in the evaluation of malnutrition and quality of life in cancer patients—A comprehensive review. Eur. J. Clin. Nutr..

[11] Thibault R., Makhlouf A.M., Mulliez A., Cristina Gonzalez M., Kekstas G., Kozjek N.R., Preiser J.C., Rozalen I.C., Dadet S., Krznaric Z. (2016). Fat-free mass at admission predicts 28-day mortality in intensive care unit patients: The international prospective observational study Phase Angle Project. Intensive Care Med..

[12] Wiech P., Dabrowski M., Bazalinski D., Salacinska I., Korczowski B., Binkowska-Bury M. (2018). Bioelectrical Impedance Phase Angle as an Indicator of Malnutrition in Hospitalized Children with Diagnosed Inflammatory Bowel Diseases-A Case Control Study. Nutrients.

[13] Mentella M.C., Scaldaferri F., Pizzoferrato M., Gasbarrini A., Miggiano G.A.D. (2019). The Association of Disease Activity, BMI and Phase Angle with Vitamin D Deficiency in Patients with IBD. Nutrients.

[14] Ryan E., McNicholas D., Creavin B., Kelly M.E., Walsh T., Beddy D. (2019). Sarcopenia and Inflammatory Bowel Disease: A Systematic Review. Inflamm. Bowel Dis..

[15] Caio G., Lungaro L., Caputo F., Zoli E., Giancola F., Chiarioni G., De Giorgio R., Zoli G. (2021). Nutritional Treatment in Crohn’s Disease. Nutrients.

[16] Galluzzo M., D’Adamio S., Pastorino R., Andreoli A., Servoli S., Bianchi L., Talamonti M. (2018). Effect of anti IL-12/23 on body composition: Results of bioelectrical impedance analysis in Caucasian psoriatic patients. Expert Opin. Biol. Ther..

[17] Feagan B.J., Sandborn W.J., Gasink C., Jacobstein D., Lang Y., Friedman J.R., Blank M.A., Johanns J., Gao L.-L., Miao Y. (2016). Ustekinumab as Induction and Maintenance Therapy for Crohn’s Disease. N. Engl. J. Med..

[18] Sands B.E., Sandborn W.J., Panaccione R., O’Brien C.D., Zhang H., Johanns J., Adedokun O.J., Li K., Peyrin-Biroulet L., Van Assche G. (2019). Ustekinumab as Induction and Maintenance Therapy for Ulcerative Colitis. N. Engl. J. Med..

[19] Macaluso F.S., Maida M., Ventimiglia M., Cottone M., Orlando A. (2020). Effectiveness and safety of Ustekinumab for the treatment of Crohn’s disease in real-life experiences: A meta-analysis of observational studies. Expert Opin. Biol. Ther..

[20] Torres J., Bonovas S., Doherty G., Kucharzik T., Gisbert J.P., Raine T., Adamina M., Armuzzi A., Bachmann O., Bager P. (2020). ECCO Guidelines on Therapeutics in Crohn’s Disease: Medical Treatment. J. Crohn’s Colitis.

[21] Baumgartner R.N., Chumlea W.C., Roche A.F. (1988). Bioelectric impedance phase angle and body composition. Am. J. Clin..

[22] Bertani L., Ribaldone D.G., Bellini M., Mumolo M.G., Costa F. (2021). Inflammatory Bowel Diseases: Is There a Role for Nutritional Suggestions?. Nutrients.

[23] Nardone O.M., de Sire R., Petito V., Testa A., Villani G., Scaldaferri F., Castiglione F. (2021). Inflammatory Bowel Diseases and Sarcopenia: The Role of Inflammation and Gut Microbiota in the Development of Muscle Failure. Front. Immunol..

[24] Schakman O., Gilson H., Thissen J.P. (2008). Mechanisms of glucocorticoid-induced myopathy. J. Endocrinol..

[25] Cabre E., Gassull M.A. (2001). Nutrition in inflammatory bowel disease: Impact on disease and therapy. Curr. Opin. Gastroenterol..

[26] Bamba S., Sasaki M., Takaoka A., Takahashi K., Imaeda H., Nishida A., Inatomi O., Sugimoto M., Andoh A. (2017). Sarcopenia is a predictive factor for intestinal resection in admitted patients with Crohn’s disease. PLoS ONE.

[27] Pedersen M., Cromwell J., Nau P. (2017). Sarcopenia is a Predictor of Surgical Morbidity in Inflammatory Bowel Disease. Inflamm. Bowel Dis..

[28] Holt D.Q., Varma P., Strauss B.J.G., Rajadurai A.S., Moore G.T. (2017). Low muscle mass at initiation of anti-TNF therapy for inflammatory bowel disease is associated with early treatment failure: A retrospective analysis. Eur. J. Clin. Nutr..

[29] Cederholm T., Barazzoni R., Austin P., Ballmer P., Biolo G., Bischoff S.C., Compher C., Correia I., Higashiguchi T., Holst M. (2017). ESPEN guidelines on definitions and terminology of clinical nutrition. Clin. Nutr..

[30] Gonzalez M.C., Barbosa-Silva T.G., Heymsfield S.B. (2018). Bioelectrical impedance analysis in the assessment of sarcopenia. Curr. Opin. Clin. Nutr. Metab. Care.

[31] Pizzoferrato M., de Sire R., Ingravalle F., Mentella M.C., Petito V., Martone A.M., Landi F., Miggiano G.A.D., Mele M.C., Lopetuso L.R. (2019). Characterization of Sarcopenia in an IBD Population Attending an Italian Gastroenterology Tertiary Center. Nutrients.

[32] Kim S.H., Kim Y.S., Lee S.H., Lee H.M., Yoon W.E., Kim S.H., Myung H.J., Moon J.S. (2021). Evaluation of nutritional status using bioelectrical impedance analysis in patients with inflammatory bowel disease. Intest. Res..

[33] Kyle U.G., Bosaeus I., De Lorenzo A.D., Deurenberg P., Elia M., Gómez J.M., Heitmann B.L., Kent-Smith L., Melchior J.C., Pirlich M. (2004). Bioelectrical impedance analysis—part I: Review of principles and methods. Clin. Nutr..

[34] Peng Z., Xu D., Li Y., Peng Y., Liu X. (2022). Phase Angle as a Comprehensive Tool for Nutritional Monitoring and Management in Patients with Crohn’s Disease. Nutrients.

[35] Subramaniam K., Fallon K., Ruut T., Lane D., McKay R., Shadbolt B., Ang S., Cook M., Platten J., Pavli P. (2015). Infliximab reverses inflammatory muscle wasting (sarcopenia) in Crohn’s disease. Aliment. Pharmacol. Ther..

[36] Emerenziani S., Biancone L., Guarino M.P.L., Balestrieri P., Stasi E., Ribolsi M., Rescio M.P., Altomare A., Cocca S., Pallone F. (2017). Nutritional status and bioelectrical phase angle assessment in adult Crohn disease patients receiving anti-TNFalpha therapy. Dig. Liver Dis. Off. J. Ital. Soc. Gastroenterol. Ital. Assoc. Study Liver.

[37] Wolk K., Mallbris L., Larsson P., Rosenblad A., Vingard E., Stahle M. (2009). Excessive body weight and smoking associates with a high risk of onset of plaque psoriasis. Acta Derm.-Venereol..

[38] Jensen P., Skov L. (2016). Psoriasis and Obesity. Dermatology.

[39] Narula N., Wong E.C.L., Dulai P.S., Marshall J.K., Colombel J.F., Reinisch W. (2021). Week 6 Calprotectin Best Predicts Likelihood of Long-term Endoscopic Healing in Crohn’s Disease: A Post-hoc Analysis of the UNITI/IM-UNITI Trials. J. Crohn’s Colitis.

[40] Bertani L., Blandizzi C., Mumolo M.G., Ceccarelli L., Albano E., Tapete G., Svizzero G.B., Zanzi F., Coppini F., de Bortoli N. (2020). Fecal Calprotectin Predicts Mucosal Healing in Patients with Ulcerative Colitis Treated with Biological Therapies: A Prospective Study. Clin. Transl. Gastroenterol..

[41] Natale E., Marsocci A. (2016). Are clinical trial results transferable in the real life?. Monaldi Arch. Chest Dis. Arch. Monaldi Mal. Torace.

[42] García-Sánchez V., Iglesias-Flores E., González R., Gisbert J.P., Gallardo-Valverde J.M., González-Galilea A., Naranjo-Rodríguez A., de Dios-Vega J.F., Muntané J., Gómez-Camacho F. (2010). Does fecal calprotectin predict relapse in patients with Crohn’s disease and ulcerative colitis?. J. Crohns Colitis.

[43] Mumolo M.G., Bertani L., Ceccarelli L., Laino G., Di Fluri G., Albano E., Tapete G., Costa F. (2018). From bench to bedside: Fecal calprotectin in inflammatory bowel diseases clinical setting. World J. Gastroenterol..

